# The cost-effectiveness of screening lung cancer patients for targeted drug sensitivity markers

**DOI:** 10.1038/bjc.2012.60

**Published:** 2012-02-28

**Authors:** A J Atherly, D R Camidge

**Affiliations:** 1 Department of Health Systems, Management and Policy, Colorado School of Public Health, University of Colorado, Aurora, CO, USA; 2 Division of Medical Oncology, University of Colorado Cancer Center, Anschutz Medical Campus, Anschutz Cancer Pavillion, Mailstop F704, 1665 North Aurora Court, Aurora 80045, CO, USA

**Keywords:** ALK, crizotinib, lung cancer, EGFR, cost-effectiveness

## Abstract

**Background:**

New oncology drugs are being developed in conjunction with companion diagnostics with approval restricting their use to certain biomarker-positive subgroups. We examined the impact of different predictive biomarker screening techniques and population enrichment criteria on the cost-effectiveness of targeted drugs in lung cancer, using ALK and crizotinib to build the initial model.

**Methods:**

Health economic modeling of cost per Quality Adjusted Life Year was based on literature review and expert opinion. The modeled population represented advanced non-small cell lung cancer (NSCLC), eligible for predictive biomarker screening with prescribing restricted to biomarker-positive patients.

**Results:**

For assays costing $1400 per person, cost per quality-adjusted life year (QALY) gained for ALK screening all advanced NSCLC, excluding treatment cost, is $106 707. This falls to $4756 when only a highly enriched population is screened (increasing biomarker frequency from 1.6 to 35.9%). However, the same enrichment involves missing 56% patients who segregate within the unscreened group. Cheaper screening tests that miss some true positives can be more cost-effective if proportional reductions in cost exceed proportion of subjects missed. Generic modeling of idealised screening assays, including treatment cost, reveals a dominant effect of screening cost per person at low biomarker frequencies. Cost-effectiveness of <$100 000 per QALY gained is not achievable at biomarker frequencies <5% (with drug costs $1–5000 per month and screening costs $600–1400 per person).

**Interpretation:**

Cost-effectiveness of oncology drugs whose prescribing is restricted to biomarker-positive subgroups should address the cost of detecting marker-positive patients. The cost of screening dominates at low frequencies and strategies to improve cost-effectiveness based on the assay cost, drug cost and the group screened should be considered in these scenarios.

In recent years, there has been excitement about ‘personalised medicine’. In oncology, this means moving away from a ‘one size fits all’ model of treating cancer by the use of identifying markers that can reliably predict who to give and not give specific drugs. Such predictive biomarkers may address aspects of host toxicity or, increasingly, of direct anti-cancer efficacy. Most examples to date have been developed to finesse clinical practice after a specific drug has been licensed (Eichelbaum *et al*, 2006; Huang and Ratain, 2009; Pao and Chmielecki, 2010). However, new drugs are starting to be developed in conjunction with companion diagnostics such that their initial approval will involve a label restricting their use to only certain marker-positive subgroups.

Although traditional cost-effectiveness analyses have focused primarily on the cost of the treatment and its benefit in the treated population, the move towards pairing new drugs with specific drug sensitivity biomarkers will introduce additional complexities into health economic analyses (Table 1). Notably, the cost-effectiveness will depend not just on the drug itself, but also on the cost-effectiveness of the accompanying screening test (de Lima Lopes *et al*, 2011). Advances in the treatment of non-small cell lung cancer (NSCLC) can be used to illustrate the impact of different factors on the cost-effectiveness of molecular screening for drug sensitivity biomarkers in oncology.

Improved understanding of the biology of NSCLC has led to a number of novel targeted agents with differential activity in specific molecular subtypes of the disease (Kwak *et al*, 2010; Pao and Chmielecki, 2010; Kris *et al*, 2011; Siegel *et al*, 2011). For example, epidermal growth factor receptor (EGFR) mutations present in ∼10% of the Western lung cancer population were retrospectively associated with maximal benefit from EGFR inhibitor drugs, after these drugs were initially licensed on the basis of minor benefit in unselected NSCLC populations (Shepherd *et al*, 2005; Pao and Chmielecki, 2010). In contrast, because it was prospectively identified, crizotinib was recently approved by the US Food and Drug Administration (FDA) only for the treatment of advanced NSCLC proven to harbour an ALK gene rearrangement, an abnormality present in <5% of the NSCLC population (Kwak *et al*, 2010).

The purpose of this paper is to examine the impact of different predictive biomarker screening techniques and enrichment criteria on the cost-effectiveness of targeted drugs in oncology, using ALK and crizotinib as the initial example. Beyond the cost per test of the predictive biomarker in the baseline screening, other factors also need to be considered when modeling the cost-effectiveness of molecular screening. First, who to screen? *ALK* rearrangements are not randomly distributed within NSCLC (Solomon *et al*, 2009). The term NSCLC covers lung cancers with several different histologies including adenocarcinoma, squamous cancer and large cell carcinoma (American Cancer Society, 2011). The majority of NSCLCs that are ALK positive exhibit adenocarcinoma histology, and are likely to occur in individuals with little or no tobacco exposure (Camidge *et al*, 2010). In addition, as the majority of adenocarcinomas exhibit only a single oncogenic driver mutation and some mutations are already commonly screened for, *ALK* rearrangements have also been noted to be more common among those who are known to be wild type for both *EGFR* and *KRAS*, two other mutations known to be involved in NSCLC (Camidge *et al*, 2010; Kris *et al*, 2011). Consequently, the ‘hit rate’ of screening with any given technique can be altered by choosing to rule in or rule out certain groups based on a series of other factors. However, as exceptions occur, enrichment also carries with it the risk of missing some true marker-positive patients because they fall into a category resulting in them not being tested for the marker at all.

For ALK, there are a number of tests that can be used to screen patients. Currently, assessment of ALK positivity using fluorescence *in-situ* hybridisation (FISH) has been the only screening criteria used in the clinical studies of crizotinib. In the recent FDA-accelerated approval submission, FISH is also the molecular test that was filed as a companion diagnostic. However, at least two other potential screening tests for ALK are being explored.

Reverse transcription–polymerase chain reaction (RT–PCR) can detect the presence of specific abnormal fusion transcripts. However, recent work suggests that RT–PCR for *EML4–ALK*, the most common ALK rearrangement in NSCLC, misses up to 30% of cases, taking *ALK* FISH-positives as the definition of ‘true’ ALK positivity (Kwak *et al*, 2010).

Immunohistochemistry (IHC) can detect the abnormal expression of the ALK protein. However, the reproducibility, sensitivity and specificity vary considerably according to the antibody, antigen retrieval and amplification systems, and the scoring method and cutpoints used (Mino-Kenudson *et al*, 2010; Camidge *et al*, 2011b).

Here we calculate the cost-effectiveness of marker screening using different assumptions about screening techniques, the population screened and the targeted treatment. On the basis of the initial ALK example, we then extended our analyses to broadly explore the factors influencing the cost-effectiveness of different molecular screening strategies for targeted anti-cancer drug sensitivity biomarkers in general.

## Materials and methods

### Methodology overview

To calculate the cost-effectiveness of predictive biomarker screening and treatment of marker-positive disease, values relating to ALK and crizotinib were determined by a review of the literature and canvassed expert opinion to establish (a) the cost of each screening test per patient, (b) the effectiveness of each screening test relative to a perceived gold standard, (c) the ability of different clinical and pathological factors to enrich for *ALK* rearrangements and (d) the benefit of treatment in patients identified as ALK-positive by the screening.

On average, crizotinib delays ALK-positive cancer progression for 9–10 months (Kwak *et al*, 2010; Camidge *et al*, 2011a). These numbers are remarkably similar to the benefits of the EGFR inhibitors, erlotinib and gefitinib, in EGFR mutant advanced NSCLC. This suggests such timelines may reflect something fundamental in the biology of oncogenically driven NSCLC in the time it takes to evolve acquired resistance to specific targeted therapies (Mok *et al*, 2009; Mitsudomi *et al*, 2010; Maemondo *et al*, 2010, Zhou *et al*, 2011). Although we do not have randomised data compared with placebo to be able to accurately attribute health gain from the intervention itself, as duration of therapy may extend beyond the time of first progression for both EGFR and ALK inhibitor therapy, we opted to consider progression-free survival (PFS) as if it truly represented drug-related health gain (Riely *et al*, 2007; Camidge *et al*, 2011a). In addition, there is considerable data on the rapid and dramatic symptomatic improvement seen when these oncogene-‘addicted’ cancers are treated with their specific inhibitors (Crino *et al*, 2011; Camidge *et al*, 2011a). Therefore, for the purposes of both the initial ALK calculation and the later generic biomarker treatment modeling, an average gain of 10 months of perfect quality of life from targeted therapy, that is, 0.83 quality-adjusted life years (QALYs), in those who are positive for a predictive biomarker was assumed.

Prices for the different tests vary depending on the payer and system. In the United States, for example, different insurers reimburse charges at different rates. To limit this complexity, we have therefore taken charges, not reimbursements as our base values. We estimated costs for pathological testing, including both technical and professional fees, utilising Medicare list prices and the associated University of Colorado charges. Costs for tissue acquisition were not included, assuming the analyses were conducted on pre-existing archival material. On the basis of the expert opinion, *ALK* FISH testing was estimated at $1400 per test and was taken as the reference standard for positivity. RT–PCR was estimated at $875 per test, but may miss up to 30% of true ALK-positive cases. A validated IHC assay was also considered likely to be cheaper than FISH testing and was estimated at $600 per test, but if used alone may miss up to 20% of true ALK-positive cases if only 3+ IHC staining (the level associated with no false positives) was used to define positivity (Paik *et al*, 2011; Yi *et al*, 2011) (Table 2).

All costs were in US dollars. In calculating costs, the societal perspective was adopted. As the median age of onset of lung cancer is ∼70, in many cases the recipients will be retired and out of the workforce (Owonikoko *et al*, 2007). Key costs were, therefore, the cost of the screening plus the treatment for those with positive tests. The estimated cost per QALY acceptability threshold used for discussion purposes was $100 000 (Cutler *et al*, 2006).

## Results

### Initial modeling of ALK screening-crizotinib treatment pairings and ALK enrichment factors

In selecting the patient population to be screened, we examined four different primary screening filters of increasing stringency.

1. All patients with advanced stage NSCLC.
2. Patients with advanced stage NSCLC who have tumours with adenocarcinoma histology.
3. Patients with advanced stage NSCLC who have tumours with adenocarcinoma histology who have never smoked.
4. Patients with advanced stage NSCLC who have tumours with adenocarcinoma histology, and who have never smoked and who are known to be both EGFR and KRAS wild type.

Our modeling calculated the impact of each of the different enrichment steps as follows:

1. *All patients with advanced stage NSCLC:* Overall, ∼4% of patients with NSCLC have been reported to harbour ALK rearrangements (Solomon *et al*, 2009). However, most of the resection cases analysed have been heavily biased towards adenocarcinoma histologies, which may predominate among the early-stage lesions present in such series. Although the proportions may be changing over time and may differ between countries, in a recent US series using the SEER database, adenocarcinoma, including bronchoalveolar and adenosquamous histologies, represented ∼39% of NSCLC (Owonikoko *et al*, 2007). In contrast, it was the dominant histology in 72% of the cases analysed for ALK across several series (Weickhardt and Camidge, 2011). Consequently, when adjusting for an expected lower frequency of adenocarcinoma that in most of the reported ALK analysis series, we projected an initial ALK-positive frequency of ∼1.6% among unselected US NSCLC cases.
2. *Patients with advanced stage NSCLC who have tumours with adenocarcinoma histology:* Across several series, 89% of ALK rearrangements in NSCLC occur in adenocarcinomas (including bronchoalveolar and adenosquamous histologies) (Weickhardt and Camidge, 2011). Combined with data on the expected frequency of adenocarcinomas in the United States, screening 39% of the population with advanced stage NSCLC should, therefore, capture 89% of ALK rearrangements (Owonikoko *et al*, 2007). This suggests an ALK-positive frequency in this population of ∼3.7%.
3. *Patients with advanced stage NSCLC who have tumours with adenocarcinoma histology who have never smoked:* Never smokers represent ∼15% of NSCLC in the West (Ramalingam *et al*, 2011). Assuming that the proportion of never smokers is constant by histological subtype, the same proportions will apply to adenocarcinomas as to NSCLC in general. Approximately 56% of ALK rearrangements in NSCLC occur in never smokers with adenocarcinoma histology (Weickhardt and Camidge, 2011). Screening the 15% of the population with advanced stage NSCLC who have tumours with adenocarcinoma histology who have never smoked should, therefore, capture 56% of the ALK rearrangements present in the adenocarcinoma population. This suggests an ALK-positive frequency in this population of ∼13.7%.
4. *Patients with advanced stage NSCLC who have tumours with adenocarcinoma histology, and who have never smoked and who are known to be both EGFR and KRAS wild type:* In the West, EGFR mutations and KRAS mutations occur in ∼25% and 17–25% of adenocarcinomas, respectively (Riely *et al*, 2008; Girard *et al*, 2011). Therefore, EGFR and KRAS wild type status exists in 75% and 75–83% of such cases, respectively. Among never smokers with adenocarcinoma, the proportion of EGFR mutations increases to ∼50% (Girard *et al*, 2011). Although the proportion of KRAS mutations does not appear to change on a statistically significant level according to smoking status, the absolute proportion does tend to be lower among never smokers, for example,∼15% (Riely *et al*, 2008). EGFR and KRAS mutations appear to be effectively 100% mutually exclusive (Girard *et al*, 2011). Therefore, in our calculations we assumed that 35% of never smokers with adenocarcinoma of the lung would be both EGFR and KRAS wild type. The coexistence of ALK positivity and either EGFR or KRAS mutations is rare, but has been reported in ∼8% of ALK FISH-positive cases (Girard *et al*, 2011; Kris *et al*, 2011). Therefore, 92% of ALK rearrangements in NSCLC are predicted to occur in patients whose tumours can be proven to be both EGFR and KRAS wild type. Screening the 35% of never smokers with adenocarcinoma of the lung who will be both EGFR and KRAS wild type should, therefore, capture 92% of the ALK rearrangements contained within the never smoking adenocarcinoma population. This suggests an ALK-positive frequency in this population of ∼35.9%.

The expected proportions of ALK positives in each of these increasingly enriched groups, the percentage of the total advanced NSCLC that each group represents, and the estimated numbers of cases found and missed within an initial starting population of 1000 patients with advanced NSCLC are shown in Table 3. Patients with a positive screening result were assumed to receive the specific inhibitor, in this case crizotinib. The cost per QALY gained for the screening alone, excluding the cost of treatment, given the different costs and performances of the screening tests and different ALK enrichment strategies are shown in Table 3.

### Hypothetical predictive biomarker screening-treatment pairings

Using the initial ALK modeling for the extent of benefit and cost of the different screening techniques, we then moved to explore more generic modeling. We calculated the hypothetical cost-effectiveness of overall predictive marker screening-treatment pairings, assuming different costs of the treatment per unit time (ranging from $10 000 to $1000 dollars per month); different costs of the screening per person ($1400–$600 per person, assuming perfect performance of the assay in detecting the true marker-positive population at all screening costs) and different underlying frequencies of true marker positivity in the population (ranging from 1 to 50%) (Table 4).

**Figure 1:**
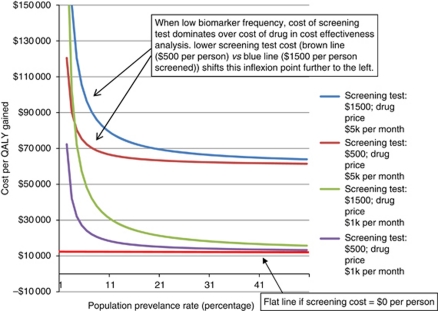
Cost per QALY gained with varying screening prices per patient, treatment prices per month and population prevalence rates of hypothetical predictive biomarker (assuming treatment of marker-positive population results in uniform gain of 0.83 QALYs per patient).

Presenting these data in a different format, we calculated the drug price necessary to achieve a cost-effectiveness ratio of $100 000 per QALY given different screening costs per person and different biomarker frequencies in the screened population (Table 5). The interrelationship between cost per QALY gained, biomarker prevalence and the effect of different screening costs per test, and of drug prices per month is presented visually in Figure 1.

## Discussion

New oncology drugs are starting to be licensed in conjunction with companion diagnostics, limiting prescribing only to those patients with maximal potential benefit from the drug. Consequently, cost-effectiveness analysis now has to address aspects of the screening as well as the traditional cost and benefit from the treatment (Table 1).

Using available information on the cost and performance of different ALK detection methods, we were able to assess the potential impact of these factors on the cost-effectiveness of biomarker screening in relation to ALK and crizotinib in NSCLC (Tables 2 and 3).

There are several assumptions and limitations associated with our work. First, we used estimated commercial charges for pathology testing derived from one institution for our analyses, limiting the direct transferability of specific values worldwide. However, the purpose of this paper is not to evaluate the cost-effectiveness of a particular test that will be applicable without additional analyses across all scenarios; instead, our purpose is to illustrate the issues that will determine the cost-effectiveness and the relative contribution of prevalence rates, screening cost, screening sensitivity and specificities, and the cost of the treatment. The precise screening cost in any given scenario will depend on a multitude of factors, including the particular provider of the test, and the health organisation and payer system involved. To generate data for modeling, we assumed that the PFS in single arm studies represented the actual health gain from the intervention and that the dramatic clinical responses seen when specific inhibitors are given to pre-selected oncogene-addicted tumours would produce a period of time with perfect quality of life. Inevitably, as either assumption weakens, the health gain from the intervention in our modeling would become correspondingly less. We assumed that the median age of lung cancer is 70-year-old, which in turn assumes that the molecular markers being screened for are uncorrelated with age. In fact, for several actionable molecular abnormalities, including ALK rearrangements, the median age of onset may be several years younger than for the general lung cancer population (Weickhardt and Camidge, 2011). Consequently, in such scenarios there may be additional health benefits that could be addressed in the form of productivity gained. We also assumed that the proportion of never smokers is constant by histological subtype in the absence of additional data (Ramalingam *et al*, 2011). However, if the never smoking rate were, in fact, >15% among adenocarcinomas, this would increase the denominator in the relevant enrichment step, increasing both the absolute costs and the cost per positive. Finally, we assume that PFS represents the dominant clinical benefit, as this is the only clinical data available in most cases; however, if overall survival is also longer then this would be an additional benefit and would reduce the cost per QALY gained.

Unlike most health economic analyses, we did not conduct a classical Markov model, comparing those who test positive and get the specific inhibitor with those who test negative and get standard chemotherapy. Instead, we focused our analyses on the pairing of the screening and the treatment, assuming that for those who test positive, after the treatment has run it's course, they simply proceed with the remaining standard therapies.

From our modeling, if all individuals with advanced NSCLC are screened for ALK positivity by FISH and the 1.6% who are found to positive are treated and gain 0.83 QALYs each, the total gain across the population will be ∼0.013 QALYs (∼5 extra days of perfect life) per individual screened. The health gain increases, on average, as each of the screening techniques is directed to populations with higher rates of ALK positivity (Table 3). The screening cost per QALY gained – omitting the treatment cost – is relatively high when screening all individuals with advanced NSCLC – $1 06 707 per QALY gained for the FISH test. However, as the population screened becomes more enriched and the mean health gain increases, the cost per QALY gained decreases. In addition, beyond increasing the screening ‘hit rate’, sequential enrichment provides further absolute cost savings through smaller and smaller proportions of the total population being screened (column 3, Table 3).

When comparing the cost-effectiveness of the different screening tests, both cost and performance of the test have to be considered. Specifically, an ‘imperfect’ test may be more cost-effective than a ‘perfect’ test, provided the proportion of cases missed remains less than the proportional reduction in the screening cost associated with using the ‘imperfect’ assay. For example, although the RT–PCR test misses 30% of cases, our estimate is that the test is ∼40% less costly ($875 *vs* $1400). Thus, the cost per QALY gained is uniformly 10% lower than for FISH testing.

When both drug and screening costs are included, in a more general oncology model that assumes perfect performance of all screening tests, Table 4 shows that at very low biomarker frequencies, the price of the screening test is enormously important in the cost-effectiveness of the overall screening-treatment pairing. In the extreme, if the frequency of the marker is 1% and the price of the screening test is $1400, a cost per QALY gained of $100 000 is not achievable at any drug price. It just becomes achievable at a drug price of $1 000 per month if the screening cost drops to $600 per person tested. However, as the underlying frequency in the population increases above a relatively low threshold (⩾5% Table 4), a cost per QALY gained of ⩽$100 000 is achievable with screening costs of $1400 per person tested when the drug is priced in the $1000–$5000 per month range.

As the frequency of the biomarker increases, the relative contribution of the screening costs to the overall cost per QALY gained, and, by extension, the effect of differences in the screening cost per person becomes correspondingly less (Tables 4 and 5). At low biomarker prevalence rates, there is a substantial difference in the drug price per month needed to achieve cost-effectiveness, which is heavily influenced by the price of the screening test per person. However, as biomarker prevalence rates increase, the drug price necessary to achieve cost-effectiveness for the differently priced screening tests converges. At a prevalence rate of 50%, the $800 difference in screening cost per person can be offset with only a $160 difference in drug price per month.

This can be seen visually in Figure 1. If the cost of screening were zero dollars per person tested, the lines in Figure 1 would be flat with only the cost of the drug affecting the cost per QALY gained. However, when there is a cost associated with screening, the graph shows that at low biomarker prevalence rates, the cost of the screening test is the critical factor in the overall cost-effectiveness of the treatment. The cost of the screening test per person (shown for $1500 and $500 per person estimates at each of two different monthly drug prices) influences the point of inflexion where biomarker frequency becomes the dominant contributor to the cost per QALY gained. Lower screening costs per person left-shift, and higher screening costs right-shift the point of inflexion. However, once the biomarker prevalence rate exceeds ∼3–5% (depending on the exact cost of the screening test per person) in Figure 1, the price of the drug treatment alone dominates. Beyond (i) the cost per person of the screening assay, (ii) the frequency of biomarker positivity in the screened population and (iii) the price of the drug, the only other factor that will affect the cost per QALY gained will be the extent of benefit from the treatment in the biomarker-positive group. For a continuously dosed drug without screening costs, extent of benefit does not influence the cost-effectiveness, as changes in the denominator (QALYs gained) are balanced by equivalent changes in the numerator (required duration of therapy that is, total cost of drug). In contrast, when screening costs occur, as these are a one-time expenditure, they only appear in the numerator. With a greater benefit from the drug, the cost per QALY, taking into account screening costs, will therefore subtly decrease. In our modeling, we assumed a standardised gain of 0.83 QALYs. For other solid tumours and targeted therapies this figure could be different. However, we should recall that not only is this the least impactful of the four factors, it is also the only one that society and health care providers will not have any control over, whereas addressing the other three factors can significantly affect the cost-effectiveness of any screening-treatment pairings in the future.

Pulling all these data together, we can make certain observations about the cost-effectiveness of screening for predictive biomarkers in oncology. First, when considering the use of cheaper but ‘imperfect’ assays, the cost of the assay and the drug, and the frequency of the biomarker have to be considered. We would suggest that, with drug and assay costs within the range we have modeled, it makes little sense to potentially miss marker-positive patients through the use of imperfect but cheaper assays, when the biomarker of interest is above a certain threshold (∼5%). This is because at higher biomarker frequencies, the difference in assay cost makes minimal difference to the overall cost-effectiveness of the screening-treatment pairing. Second, for rare subtypes of a disease, in our model existing at <5% of the population, acceptable cost-effectiveness may only be achieved through either reducing the screening price or through adopting clinical enrichment strategies to narrow the focus to populations in which the biomarker exists at higher levels (Table 5).

The development of multiplexed assays may be one way to achieve the equivalent of lower screening costs per positive, without the risks of missing true positives through focused clinical enrichment policies. Although the frequency of each individual marker may still be low, tests such as the SNaPshot or sequenome assays used by the Lung Cancer Mutation Consortium can check for mutations in multiple different genes at the same time (Kris *et al*, 2011). Using these assays, for the cost of one screening test, information may be generated on enough different mutations to produce a hit rate for ‘actionable biomarkers’ of >40% in some NSCLCs, well above the level needed to avoid the screening costs being dominant in our modeled cost-effectiveness analysis. In our modeling, such multiplexing would effectively shift the cost per QALY calculation to the lower rows in Table 4 and right-shift the curves in Figure 1. With the advent of newer technologies, such as next generation sequencing, there may even be the potential to combine information on mutations, gene rearrangements and gene copy number, together within a single multiplexed platform in the future.

With regard to clinical enrichment, it is important to recognise that no enrichment step is perfect, and some true positives will be missed simply because they exist in subgroups that are not screened. For example, in columns 4 and 5 of Table 3, although a policy of only screening patients with adenocarcinoma who had never smoked will produce an ALK positivity rate of 13.7%, in our modeling this approach would leave half of the total ALK-positive cases undiagnosed.

Lowering drug costs will also improve the cost-effectiveness. However, a higher price for drugs with small market size is a precedent already partially established by some cost-effectiveness bodies (Rawlins and Culyer, 2004), the logic being that the overall impact on society (cost × number of cases) will be low, whereas the gain for the individuals affected may be great. In addition, a higher permissible pricing may act as a partial incentive for the pharmaceutical industry to develop drugs for rare indications, as the incidence and prevalence of these newly discovered molecular subtypes of common cancers approaches that of traditional orphan diseases. Although given the protracted clinical benefit of targeted drugs in biomarker-positive patients compared with the treatment of unselected populations, the details of how and when to offer incentives may need to be updated in the era of molecular screening for drug sensitivity biomarkers in oncology.

## Figures and Tables

**Table 1:** Comparison of traditional cost-effectiveness analysis and cost-effectiveness analysis addressing molecular screening that permits personalised therapy

*Traditional cost-effectiveness analysis in oncology (unselected population)*
Cost of treatment per unit time
Life years gained from treatment before progression in unselected population
Utility of life years gained from treatment in unselected population

*Cost-effectiveness analysis in oncology addressing personalised therapy*
Cost of treatment per unit time
Cost of screening test for marker-positive population per person screened
True frequency of positivity in screened population
Missed positives existing in unscreened populations
Performance of screening test in detecting only true marker-positive population
Performance of screening test in detecting all of true marker-positive population
Life years gained from treatment before progression in marker-positive population
Utility of life years gained from treatment in marker-positive population

**Table 2:** Costs associated with different screening tests and their ability to detect true positives with 100% specificity (anaplastic lymphoma kinase-estimated example)

**Item**	**Estimated unit cost**	**Effectiveness relative to FISH (%)**
Validated FISH	$1400	100
Validated RT–PCR	$875	70
Validated IHC assay (3+ cutpoint only)	$600	80

Abbreviations: FISH=fluorescence *in-situ* hybridisation; IHC=immunohistochemistry; RT–PCR=reverse transcription–PCR.

**Table 3:** ALK=anaplastic lymphoma kinase; Enrichment strategies for ALK-positive NSCLC and the QALYs gained under different screening criteria (frequency of positive cases, cost of screening per person and performance of screening test), excluding the cost of treatment

**Screening criteria**	**Proportion of ALK+ (true value)^a^ (%)**	**% of total initial population screened (%)**	**ALK+ cases found from initial 1 000 screens**	**ALK+ cases missed from initial 1 000 screens**	**QALYs gained per person screen, FISH**	**QALYs gained per person screen, RT–PCR**	**QALYs gained per person screen, IHC^b^**	**Screening cost per QALY gained, FISH**	**Screening cost per QALY gained, RT–PCR**	**Screening cost per QALY gained, IHC^a^**
Advanced NSCLC	1.6	100	16	0	0.013	0.009	0.010	$106 707	$95 274	$57 165
Advanced stage adenocarcinoma	3.7	39	14	2	0.030	0.021	0.024	$46 144	$41 200	$24 720
Advanced stage adenocarcinoma/never smokers	13.7	5.80	8	8	0.112	0.078	0.089	$12 462	$11 127	$6676
Advanced stage adenocarcinoma/never smokers/EGFR and KRAS wild type	35.9	2	7	9	0.294	0.206	0.235	$4756	$4246	$2548

Abbreviations: ALK=anaplastic lymphoma kinase; EGFR=epidermal growth factor receptor; FISH=fluorescence *in-situ* hybridisation; IHC=immunohistochemistry; NSCLC=non-small cell lung cancer; QALYs=quality-adjusted life years; RT–PCR=reverse transcription–PCR.

a Using FISH as the gold standard.

b Using 3+ IHC cutpoint for ALK positivity.

**Table 4:** Impact of frequency of hypothetical predictive biomarker, cost of screening test per person and cost of drug per month on overall cost per QALY gained^a^

**Frequency in screened population (%)**	**Screening cost per person=$1400^b^**	**Screening cost per person=$600^b^**	**Cost per QALY gained (screening=$1400 per person, drug=$10 000 per month)**	**Cost per QALY gained (screening=$600 per person, drug=$10 000 per month)**	**Cost per QALY gained (screening=$1400 per person, drug=$5000 per month)**	**Cost per QALY gained (screening=$600 per person, drug=$5000 per month)**	**Cost per QALY gained (screening=$1400 per person, drug=$1000 per month)**	**Cost per QALY gained (screening=$600 per person, drug=$1000 per month)**
1	$140 000	$60 000	$289 157	$192 771	$228 916	$132 530	$180 723	$84 337
5	$28 000	$12 000	$154 217	$134 940	$93 976	$74 699	$45 783	$26 506
10	$14 000	$6000	$137 349	$127 711	$77 108	$67 470	$28 916	$19 277
20	$7000	$3000	$128 916	$124 096	$68 675	$63 855	$20 482	$15 663
30	$4667	$2000	$126 104	$122 892	$65 863	$62 651	$17 671	$14 458
40	$3500	$1500	$124 699	$122 289	$64 458	$62 048	$16 265	$13 855
50	$2800	$1200	$123 855	$121 928	$63 614	$61 687	$15 422	$13 494

Abbreviation: QALY=quality-adjusted life year.

a Assuming treatment of marker-positive population results in uniform gain of 0.83 QALYs per patient.

b Excluding drug costs.

**Table 5:** Drug price per month necessary to achieve a cost-effectiveness (CE) ratio of $100 000 per QALY as frequency of predictive biomarker and cost of screening per person varies^a^

**Biomarker frequency in screened population (%)**	**Monthly drug prices needed for $100 000 CE ratio when screening cost=$1400 per person**	**Monthly drug prices needed for $100 000 CE ratio when screening cost=$600 per person**
1	N/A	$2300
5	$5500	$7100
10	$6900	$7700
20	$7600	$8000
30	$7833	$8100
40	$7950	$8150
50	$8020	$8180

Abbreviation: QALY=quality-adjusted life year.

a Assuming treatment of marker-positive population results in uniform gain of 0.83 QALYs per patient.
